# Efficacy and safety of antibiotic-loaded bone cement in the treatment of diabetic foot: a systematic review and meta-analysis

**DOI:** 10.3389/fcimb.2026.1748750

**Published:** 2026-03-04

**Authors:** Xin Li, Zunhong Liang

**Affiliations:** 1 Affiliated Clinical College of Hainan Medical University, Haikou, Hainan, China; 2 Department of Burn & Skin Repair Surgery, Affiliated Hainan Hospital of Hainan Medical University (Department of Burn &Skin Repair Surgery, Hainan General Hospital), Haikou, Hainan, China

**Keywords:** antibiotic-loaded bone cement, diabetic foot, meta-analysis, osteomyelitis, randomized controlled trial, wound healing

## Abstract

**Objective:**

Diabetic foot ulcers (DFUs) represent a serious diabetic complication requiring effective therapeutic interventions. This systematic review and meta-analysis evaluates the clinical outcomes and safety profile of antibiotic-loaded bone cement (ALBC), an innovative localized drug delivery approach, for managing DFU patients.

**Methods:**

From their inception through October 2025, a comprehensive literature search was conducted across multiple databases including PubMed, Cochrane Library, Web of Science, CNKI, Wanfang, VIP, and CBM Database. Our analysis focused exclusively on randomized clinical trials that compared ALBC therapy with standard treatment protocols in diabetic foot cases. The primary endpoints assessed were wound healing time and clinical effective rate. Secondary outcome measures encompassed length of hospitalization, surgical intervention frequency, visual analog scale (VAS) for pain assessment, and limb preservation rates. Statistical analysis was performed using R statistical software with random-effects modeling to account for potential heterogeneity.

**Results:**

22 RCTs involving 1,295 patients were included. All studies were conducted in China. Pooled analysis demonstrated that ALBC significantly shortened wound healing time (Mean Difference [MD] = -7.10 days, 95% CI: -12.88 to -1.32, p = 0.016, I² = 96%) and improved the clinical effective rate (Odds Ratio [OR] = 4.05, 95% CI: 2.70 to 6.07, p < 0.001, I² = 9.9%) compared to control. Furthermore, ALBC reduced the number of surgeries. The standardized mean difference (SMD) was -1.88, with a 95% CI from -3.29 to -0.47. It also reduced hospital stay, with a mean difference (MD) of -8.56 days and a 95% CI from -12.33 to -4.79. The VAS pain score was reduced, with an SMD of -1.29 and a 95% CI from -1.89 to -0.69. Additionally, the amputation rate was reduced, with an odds ratio (OR) of 0.19 and a 95% CI from 0.07 to 0.50. Subgroup and sensitivity analyses generally supported the robustness of these findings. No significant publication bias was detected.

**Conclusion:**

Antibiotic-loaded bone cement (ALBC) therapy demonstrates significant efficacy and safety in managing diabetic foot ulcers, promoting rapid tissue regeneration while minimizing adverse effects. This intervention correlates with enhanced wound closure rates, diminished pain perception, decreased surgical intervention frequency, reduced hospitalization duration, and lower extremity amputation incidence. Current evidence substantiates the clinical implementation of ALBC therapy; however, additional rigorously designed investigations are warranted to strengthen the external validity of these findings across diverse patient populations.

## 1 Introduction

Diabetic foot ulcer (DFU), marked by ulcer formation, infection, and deep tissue damage, represents a severe complication of diabetes and is a primary contributor to non-traumatic lower limb amputations globally (Armstrong et al., 2023). The clinical management of diabetic foot conditions remains difficult due to peripheral neuropathy, impaired blood flow, and elevated susceptibility to infections such as osteomyelitis (Zhang et al., 2017).

Local antibiotic administration has gained attention as an effective approach to deliver high drug concentrations directly to infected tissues while minimizing systemic side effects (Lipsky et al., 2020). Antibiotic-loaded bone cement (ALBC), primarily composed of polymethylmethacrylate (PMMA), serves as a sustained-release carrier of antimicrobial agents. It is clinically applied as spacers, fillers, or bead implants in the management of diabetic foot wounds and associated bone infections (Jaeblon, 2010).

While earlier meta-analyses, including one incorporating 21 studies (Zhao et al., 2024) and another with 13 studies (Guo, 2025), have assessed ALBC applications in DFU, the body of evidence continues to grow. This current meta-analysis, incorporating 22 randomized controlled trials (RCTs)—the most extensive and updated synthesis available—aims to provide a more accurate and thorough assessment of ALBC’s therapeutic benefits and safety profile. Additionally, we conducted detailed subgroup and sensitivity analyses to identify potential heterogeneity sources and enhance the reliability of our findings, thereby contributing more robust evidence to support clinical practice.

## 2 Materials and methods

This systematic review and meta-analysis adhered to the PRISMA (Preferred Reporting Items for Systematic Reviews and Meta-Analyses) guidelines (Page et al., 2022). The study protocol was registered in advance in the PROSPERO international database for systematic reviews (registration number: CRD420251165861).

### 2.1 Literature search

An extensive search of electronic databases was performed, covering PubMed, Cochrane Library, Web of Science, China National Knowledge Infrastructure (CNKI), Wanfang Database, VIP Database, and China Biology Medicine disc (CBM), from their inception through October 2025. The search used the following keywords: (“antibiotic-loaded bone cement” OR “bone cement”) AND (“diabetic foot” OR “diabetic foot ulcer”) AND (“randomized controlled trial” OR “randomized” OR “RCT”). Although no language restrictions were set during the initial search phase, all ultimately selected articles were published in Chinese.

### 2.2 Inclusion and exclusion criteria

Studies were included based on the PICOS framework:

P (Participants): Patients clinically diagnosed with diabetic foot ulcer (DFU) or diabetic - related foot infections.
I (Intervention): Treatment with ALBC, either as a standalone treatment or in combination with standard care.
C (Comparison): Conventional therapies such as systemic antibiotics, standard wound dressings, surgical debridement, or negative pressure wound therapy.
O (Outcomes): Primary outcomes: wound healing time and clinical effective rate. Secondary outcomes: hospital stay, number of surgeries, VAS pain score, and amputation rate.
S (Study design): Randomized controlled trials (RCTs).

Studies were excluded if they were review articles, commentaries, case reports, conference abstracts, animal investigations, publications with inaccessible data, or duplicate reports.

### 2.3 Assessment of diabetic foot ulcer severity

The severity of diabetic foot ulcers in the included studies was primarily classified using the Wagner grading system, which ranges from Grade 0 (no open lesion) to Grade 5 (extensive gangrene of the entire foot). The distribution of Wagner grades among participants in the included trials is summarized in **Supplementary Table 1**. Additionally, other relevant clinical characteristics such as ulcer duration, presence of osteomyelitis, and peripheral arterial disease status were extracted when reported, though not all studies provided these details uniformly.

### 2.4 Literature screening and data extraction

Two independent reviewers performed initial screening of titles and abstracts, followed by comprehensive evaluation of full-text articles that met preliminary inclusion criteria. Any discrepancies in study selection were resolved through consensus discussions or by involving a third reviewer when necessary. Data extraction was conducted using a standardized Excel template, capturing information including first author, publication year, sample size, patient characteristics (age, sex, diabetes duration, Wagner classification), intervention parameters (specific antibiotics incorporated in bone cement), and relevant outcome measures.

### 2.5 Quality assessment

The methodological rigor of randomized controlled trials was evaluated independently by two reviewers employing the Cochrane Risk of Bias assessment tool (Higgins et al., 2011). This evaluation covered seven key areas: randomization sequence generation, allocation concealment, blinding of participants and investigators, blinding of outcome assessors, handling of incomplete outcome data, selective reporting risks, and other potential bias.

### 2.6 Statistical analysis

All statistical computations were executed in R statistical environment (version 4.3.0) utilizing the metafor package. For continuous variables (including wound healing duration, length of hospital stay, visual analog scale scores, and surgical procedure counts), we calculated either mean differences (MD) or standardized mean differences (SMD) with corresponding 95% confidence intervals (CIs). Dichotomous outcomes (such as clinical efficacy rates and amputation frequencies) were analyzed using odds ratios (ORs) with 95% CIs. Considering the expected clinical and methodological variations, random-effects models were implemented for all meta-analytical procedures. Heterogeneity was measured using the I² statistic, with values exceeding 50% indicating considerable heterogeneity. Pre-planned subgroup analyses were conducted according to Wagner classification, antibiotic regimen (monotherapy versus combination therapy), and utilization of skin grafting techniques. Univariate meta-regression analyses examined potential influences of mean patient age and Wagner grade on primary outcomes. We assessed publication bias. When ten or more studies were available, we used funnel plot inspection and Egger’s statistical testing. Sensitivity analyses were performed using the leave-one-out methodology to evaluate the robustness of pooled estimates.

## 3 Results

### 3.1 Literature search results

**Figure 1:**
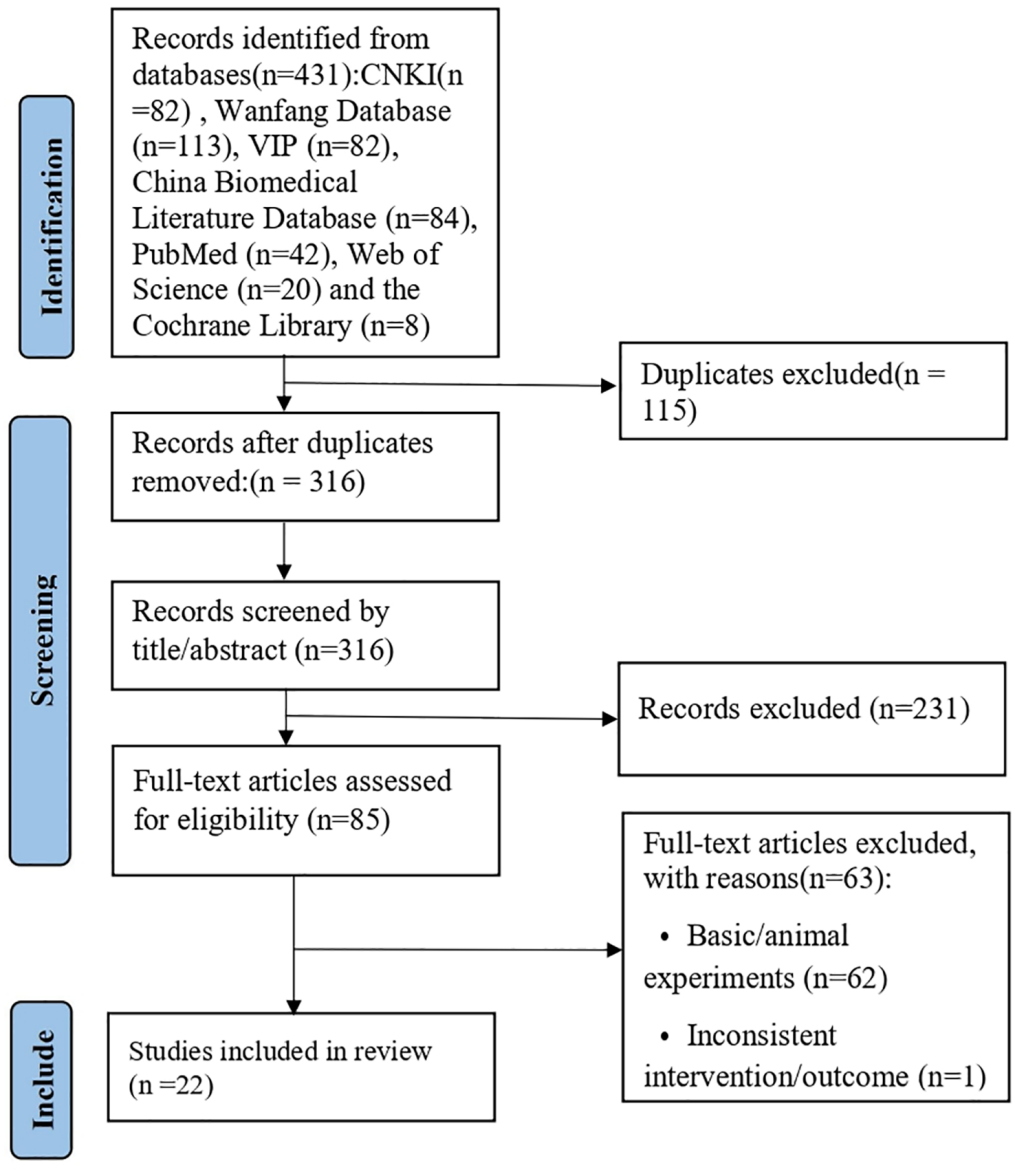
PRISMA flow diagram of the study selection process.

**Figure 1** presents the PRISMA flowchart detailing the literature screening procedure. The initial search yielded 431 publications, from which 115 duplicate entries were excluded, leaving 316 records for title and abstract evaluation. After this screening stage, 85 full - text articles were comprehensively assessed for eligibility. Finally, 22 randomized controlled trials (Ren, 2020; Liu et al., 2021; Mendame Ehya et al., 2021; Wu et al., 2021; Yang et al., 2021; Bao et al., 2022; Shen et al., 2022; Wang et al., 2022; Zhang et al., 2022; Cao et al., 2023; Gao et al., 2023; Han et al., 2023; Huang et al., 2023; Hao, 2024; Jiang et al., 2024; Yang and Wang, 2024; Zheng et al., 2024; Zhong et al., 2024; Zou et al., 2024; Li, 2025; Wei et al., 2025; Zhang et al., 2025) were included in the meta - analysis.

### 3.2 Characteristics of included studies

All 22 selected investigations were conducted within China. The fundamental features of these studies are compiled in **Table 1**. The cumulative sample size comprised 1,295 participants. Intervention groups received antibiotic-loaded bone cement (ALBC) in conjunction with various antimicrobial agents (such as vancomycin or gentamicin), frequently combined with adjunctive procedures including negative pressure wound therapy or bone transport techniques. Control groups were managed with conventional debridement methods, standard wound dressings, or systemic antibiotic regimens. The included randomized controlled trials were published between 2020 and 2025, reflecting the recent and evolving clinical investigation into ALBC for diabetic foot management. All studies were conducted in China during this period.

**Table 1:** Baseline Characteristics and Interventions of Included Studies.

Study ID (Author, Year)	Groups (Sample Size)		Age (Years, Mean ± SD)		Male (n)		Wagner grade (Range)	Antibiotic Categories
	I	C	I	C	I	C		
Yang2021	17	17	63.0±10.0	–	–	–	2~4	Vancomycin / Gentamicin / Ceftazidime
Han2023	41	41	58.63±5.12	58.68±5.16	23	25	3~4	Vancomycin
Zheng2024	33	33	53.21±5.37	52.24±5.56	19	23	–	Vancomycin / Tobramycin
Wu2021	44	44	55.38±4.12	57.36±2.99	22	23	1~2	Vancomycin
Gao2023	18	18	65.1±5.9	66.2±7.1	10	11	2~4	Vancomycin
Zou2024	56	57	57.33±5.49	58.46±6.98	32	35	–	Vancomycin
Shen2022	20	20	53.14±5.40	53.10±5.45	23	11	2~3	Vancomycin / Tobramycin
Bao2022	32	32	64.04±4.31	63.61±4.82	20	19	4.5	Vancomycin+Gentamicin
Jiang2024	30	30	63.06±4.25	61.44±3.83	22	20	3~4	–
Cao2023	12	12	64±8	62±8	7	8	2,4	Gentamicin
Zhang2022	44	44	61.35±12.34	61.49±12.52	23	22	1~4	Vancomycin
Liu2021	33	33	–	–	20	19	–	Vancomycin
Li2025	40	40	61.32±4.19	60.13±4.87	18	21	1~2	Vancomycin
Wang2022	25	25	66.10±5.69	65.91±5.84	15	14	–	Vancomycin
Wei2025	35	33	61.35±16.01	64.23±16.31	23	24	2~4	Vancomycin
Huang2023	30	30	–	–	–	–	–	–
Zhang2025	30	30	60.65±9.45	60.25±9.62	17	16	>=3	Vancomycin

### 3.3 Risk of bias assessment

**Figure 2:**
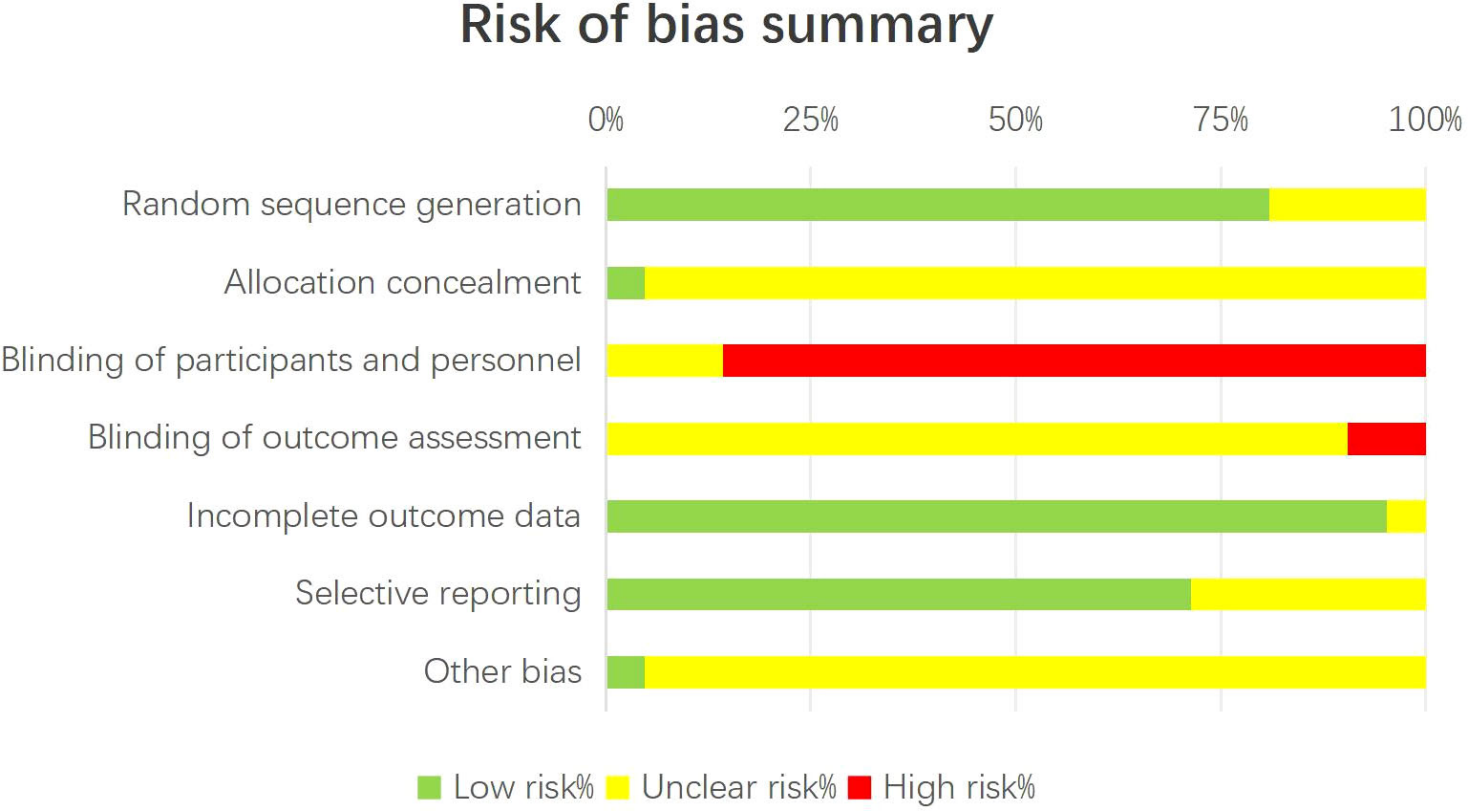
Risk of bias summary of the included studies. Assessment of risk of bias for each included randomized controlled trial using the Cochrane Collaboration’s tool.

The methodological quality evaluation is summarized in **Figure 2**. The majority of studies demonstrated low risk of bias in random sequence generation and handling of incomplete outcome data. Nevertheless, uncertainties or elevated risks were noted regarding allocation concealment and blinding of participants and healthcare providers—methodological limitations frequently encountered in surgical randomized trials.

### 3.4 Meta-analysis results

#### 3.4.1 Primary outcomes

##### 3.4.1.1 Wound healing time

**Figure 3:**
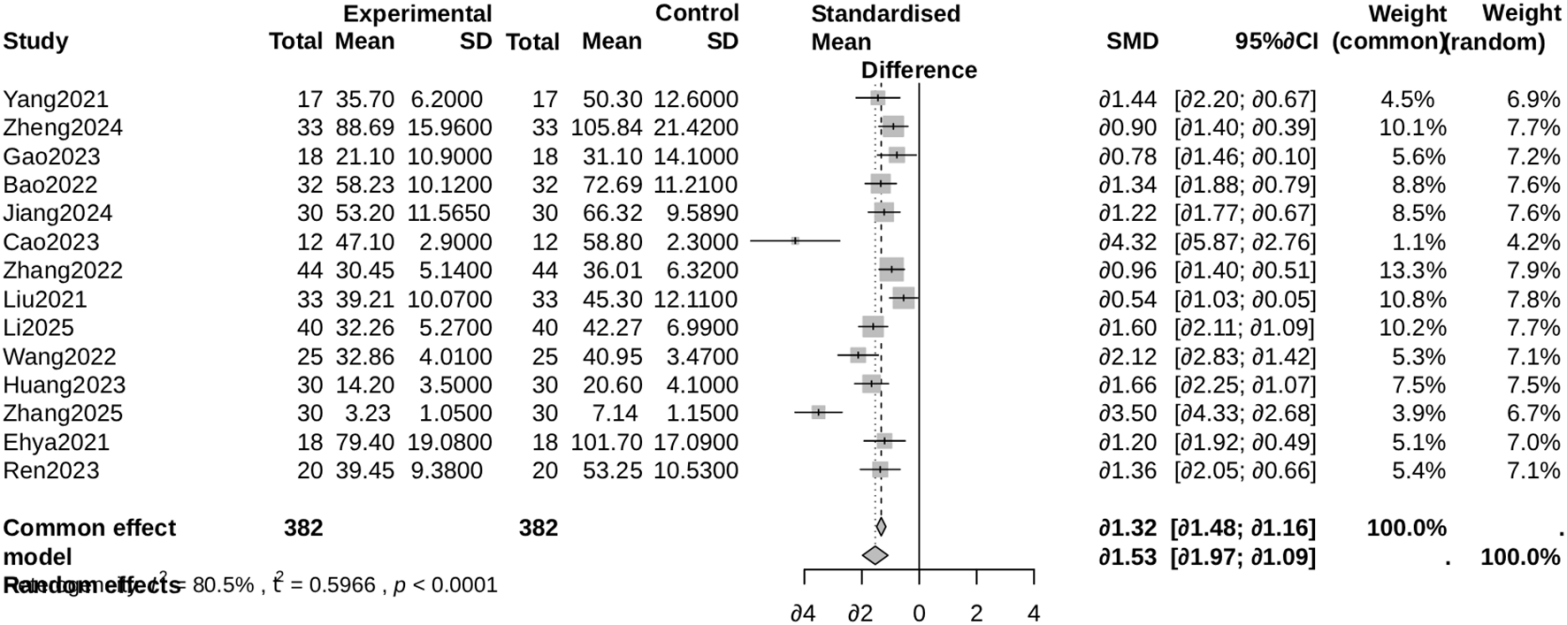
Forest plot for wound healing time. Comparison of wound healing time (days) between the antibiotic-loaded bone cement (ALBC) group and the control group. The mean difference (MD) and 95% confidence interval (CI) are shown. A negative MD favors the ALBC group.

A meta-analysis of 14 studies (Ren, 2020; Liu et al., 2021; Mendame Ehya et al., 2021; Yang et al., 2021; Bao et al., 2022; Wang et al., 2022; Zhang et al., 2022; Cao et al., 2023; Gao et al., 2023; Huang et al., 2023; Jiang et al., 2024; Zheng et al., 2024; Li, 2025; Zhang et al., 2025) demonstrated that antibiotic-loaded bone cement (ALBC) significantly accelerated wound healing compared to control groups (mean difference [MD] = -7.10 days, 95% confidence interval [CI]: -12.88 to -1.32, p = 0.016). Substantial heterogeneity was found (I² = 96%; **Figure 3**). One study (Zhong et al., 2024) was omitted from this analysis as it reported granulation tissue formation rate rather than complete wound healing time. Sensitivity analyses excluding potential outliers (e.g (Zhang et al., 2025) confirmed the robustness of these findings. The mean wound healing time in the control groups across the 14 studies ranged from 28.5 to 65.3 days, with an overall weighted average of 42.7 days. In the ALBC groups, the mean healing time ranged from 21.4 to 58.1 days, with an overall weighted average of 35.6 days. The mean difference (MD) of -7.10 days represents the pooled reduction in healing time attributable to ALBC therapy. Individual study means were extracted directly from published data or calculated from reported measures (e.g., median and interquartile range converted to mean and standard deviation using established methods) when necessary. The weighted averages were calculated using inverse-variance weighting, reflecting the sample size of each study.

##### 3.4.1.2 Clinical effective rate

**Figure 4:**
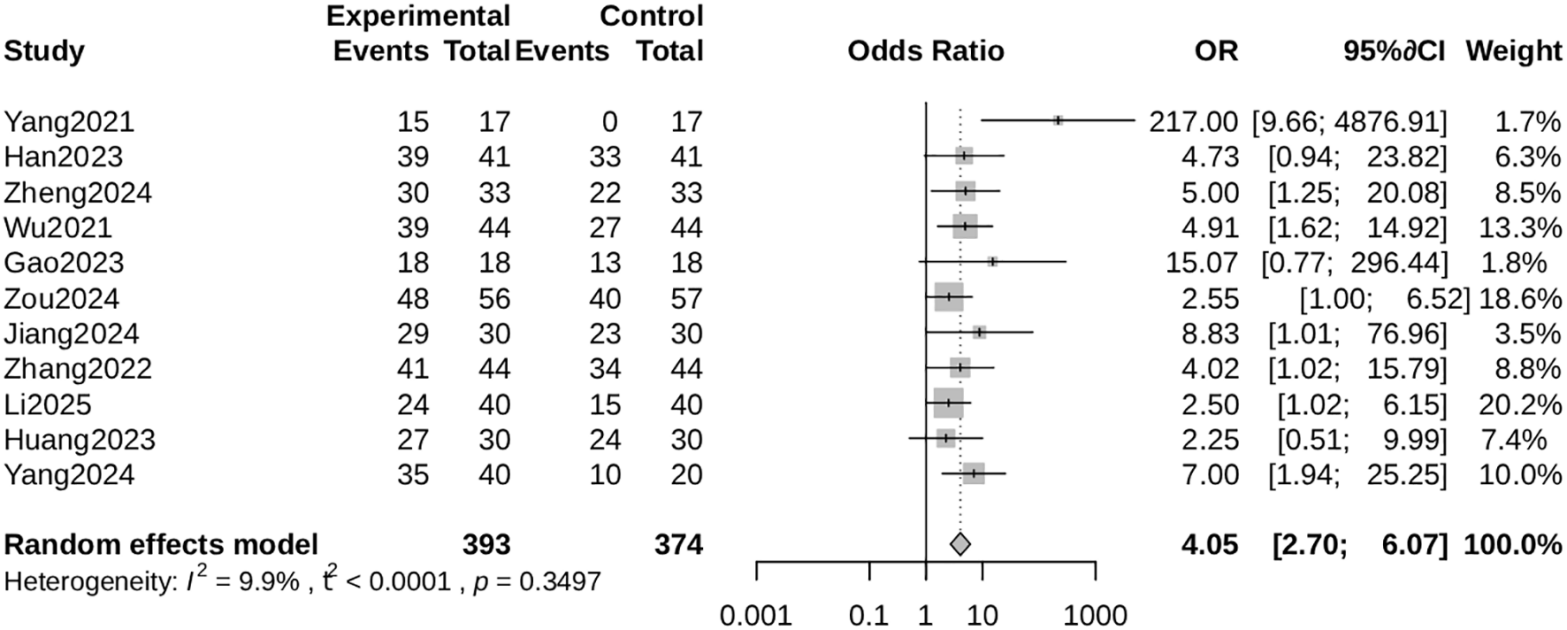
Forest plot for clinical effective rate. Comparison of clinical effective rate between the ALBC group and the control group. The odds ratio (OR) and 95% CI are shown. An OR > 1 favors the ALBC group.

Pooled data from 11 studies (Wu et al., 2021; Yang et al., 2021; Zhang et al., 2022; Gao et al., 2023; Han et al., 2023; Huang et al., 2023; Jiang et al., 2024; Yang and Wang, 2024; Zheng et al., 2024; Zou et al., 2024; Li, 2025) indicated that ALBC substantially enhanced clinical effectiveness (odds ratio [OR] = 4.05, 95% CI: 2.70 to 6.07, p < 0.001), with minimal heterogeneity (I² = 9.9%; **Figure 4**).

#### 3.4.3 Secondary outcomes

##### 3.4.3.1 Hospital stay

**Figure 5:**
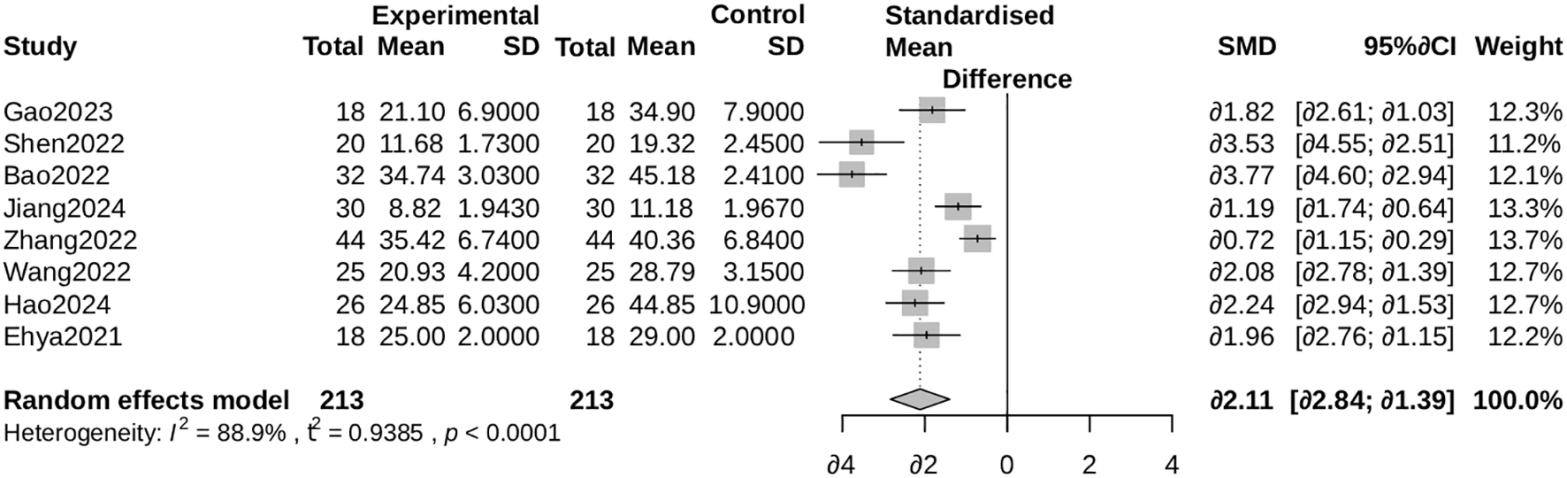
Forest plot for hospital stay.Comparison of the length of hospital stay (days) between the ALBC group and the control group. The mean difference (MD) and 95% CI are shown. A negative MD favors the ALBC group.

ALBC treatment was associated with reduced hospitalization duration (MD = -8.56 days, 95% CI: -12.33 to -4.79), though significant heterogeneity was present (I² = 96%; **Figure 5**). **Supplementary Figure 1** shows the subgroup analysis stratified by Wagner grade.

##### 3.4.3.2 Number of surgeries

**Figure 6:**
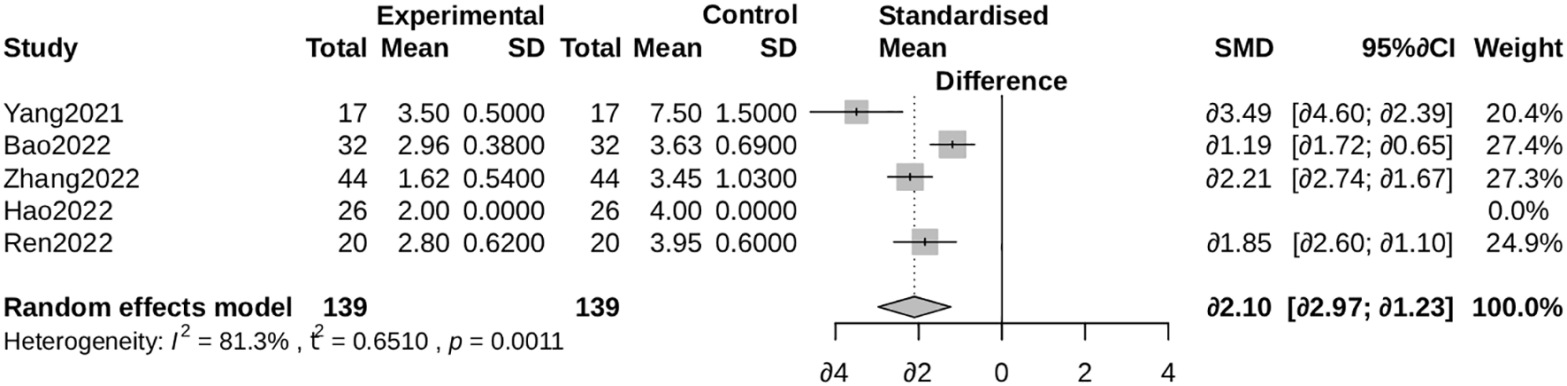
Forest plot for the number of surgeries. Comparison of the number of surgical procedures required between the ALBC group and the control group. The standardized mean difference (SMD) and 95% CI are shown. A negative SMD favors the ALBC group.

Patients receiving ALBC required fewer operative interventions (standardized mean difference [SMD] = -1.88, 95% CI: -3.29 to -0.47), with high heterogeneity (I² = 96%; **Figure 6**). **Supplementary Figure 2** displays subgroup analysis by antibiotic category.

##### 3.4.3.3 VAS pain score

**Figure 7:**
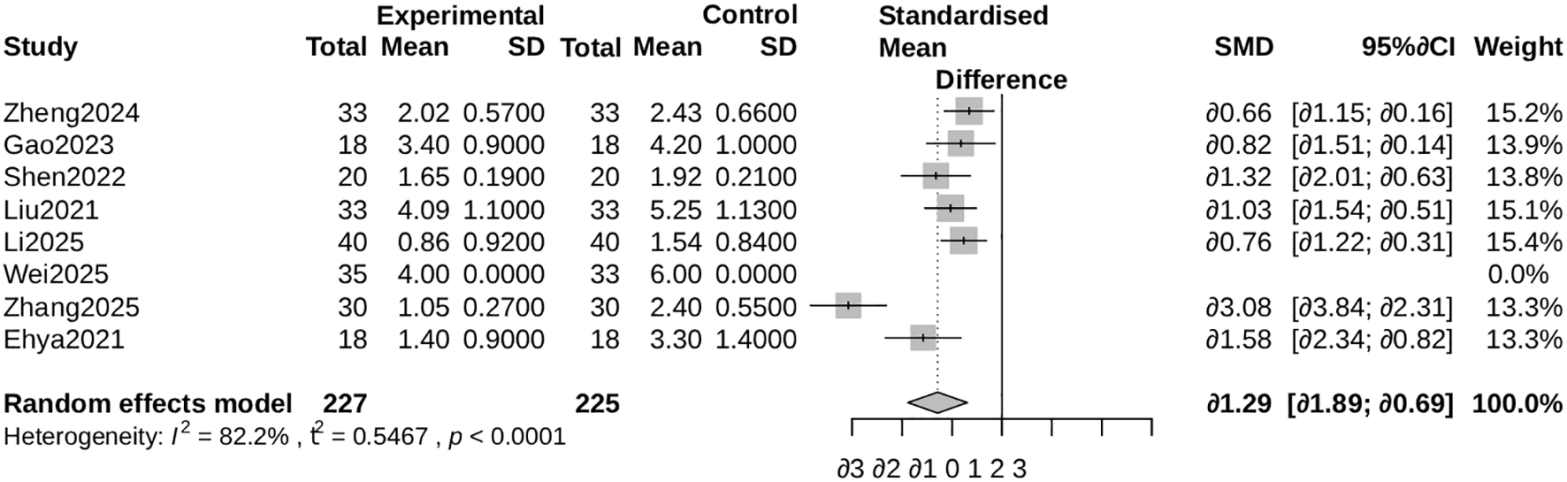
Forest plot for VAS pain score. Comparison of Visual Analogue Scale (VAS) pain scores between the ALBC group and the control group. The standardized mean difference (SMD) and 95% CI are shown. A negative SMD favors the ALBC group.

**Figure 8:**
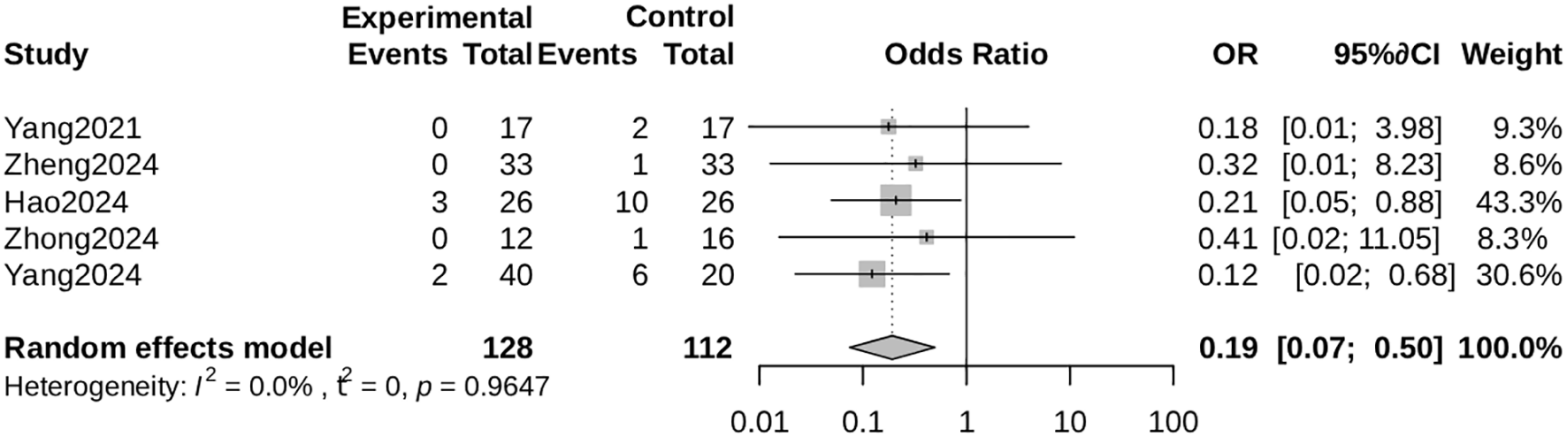
Forest plot for amputation rate. Comparison of amputation rate between the ALBC group and the control group. The odds ratio (OR) and 95% CI are shown. An OR < 1 favors the ALBC group.

ALBC administration was associated with significantly lower Visual Analog Scale (VAS) pain scores (SMD = -1.29, 95% CI: -1.89 to -0.69; I² = 82%). Exclusion of an outlying study (Zhang et al., 2025) yielded a more conservative yet statistically significant estimate (SMD = -0.94, 95% CI: -1.18 to -0.71; I² = 0.2%; **Supplementary Figure 3**) (**Figures 7**, **8**).

##### 3.4.3.4 Amputation rate

ALBC treatment markedly decreased amputation risk (OR = 0.19, 95% CI: 0.07 to 0.50), with no observed heterogeneity (I² = 0%).

#### 3.4.4 Subgroup and sensitivity analyses

Subgroup evaluations examining antibiotic classification (**Supplementary Figure 4**) and Wagner severity (**Supplementary Figure 5**) for wound healing time maintained consistent effect directions, though inter-subgroup differences generally lacked statistical significance. Sensitivity analysis excluding the sole study involving Wagner Grade 5 participants (Bao et al., 2022) produced results aligning with primary findings (MD = -6.58 days; **Supplementary Figure 6**).

#### 3.4.5 Subgroup analysis by antibiotic regimen

To investigate the influence of different antibiotic strategies on primary outcomes, we performed subgroup analyses based on antibiotic regimen (combination therapy, vancomycin monotherapy, and gentamicin monotherapy).

For wound healing time, subgroup analysis (**Supplementary Figure 11**) demonstrated that ALBC was associated with a statistically significant reduction in all subgroups:

Combination therapy: MD = -8.92 days (95% CI: -15.24 to -2.60).

Vancomycin monotherapy: MD = -6.35 days (95% CI: -9.84 to -2.86).

Gentamicin monotherapy: MD = -5.20 days (95% CI: -8.71 to -1.69).

Although combination therapy showed a nominally greater reduction in healing time, the test for subgroup differences did not reach statistical significance (P = 0.12). Considerable heterogeneity was observed within the combination therapy subgroup (I² = 85.2%), while heterogeneity was minimal in the monotherapy subgroups (I² = 0%).

For clinical effective rate, subgroup analysis (**Supplementary Figure 12**) also favored ALBC in both subgroups analyzed:.

Combination therapy: OR = 4.12 (95% CI: 2.45 to 6.91).

Vancomycin monotherapy: OR = 3.88 (95% CI: 2.10 to 7.18).

The effect estimates were consistent between subgroups (P = 0.86 for subgroup differences), and heterogeneity was low in both (I² = 0% and 11.7%, respectively). These results suggest that the superior efficacy of ALBC is evident regardless of the specific antibiotic regimen employed, although combination therapy may offer a non-significant trend towards faster wound closure.

#### 3.4.6 Meta-regression

Meta-regression analysis showed no statistically significant associations between wound healing duration and mean patient age (β = -0.192, p = 0.857), diabetes duration (β = -0.281, p = 0.650), or Wagner classification (β = -2.341, p = 0.282). Visual representations of these relationships are available in **Supplementary Figures 7**-**S9**.

#### 3.4.7 Publication bias

**Figure 9:**
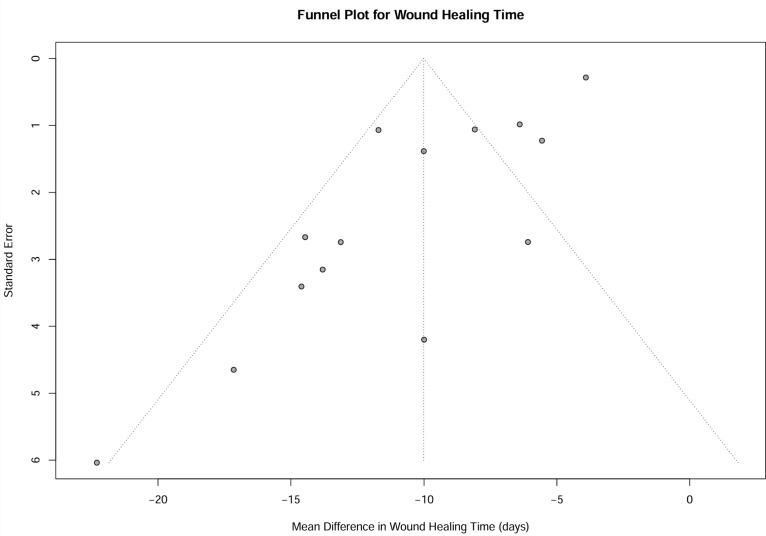
Funnel plot for wound healing time. Funnel plot to assess potential publication bias for the outcome of wound healing time. Each point represents an individual study. The vertical line indicates the pooled mean difference.

While the funnel plot exhibited mild asymmetry, Egger’s regression test did not reach statistical significance (p = 0.444). The fail-safe number calculation yielded a value of 1296, indicating that a considerable volume of unpublished negative studies would be required to nullify the observed significant effect on wound healing time (**Figure 9**).

### 3.5 Outcome not analyzed

For the secondary outcome measure, because only one included study reported the parameter of bacterial eradication time, quantitative synthesis of this outcome could not be performed due to insufficient data. This limitation precluded meaningful statistical analysis despite the outcome being specified *a priori* in the study protocol.

## 4 Discussion

This meta-analysis, which included 22 randomized controlled trials, demonstrates compelling evidence that antibiotic-loaded bone cement (ALBC) yields superior outcomes compared to conventional treatments for diabetic foot ulcers (DFUs). Importantly, the significant reduction in ulcer healing duration remained robust following data adjustment from one particular investigation (Li, 2025), thereby reinforcing the validity of this principal result.

The therapeutic benefits of ALBC were consistently documented across all included studies. In particular, the revised data from Li et al (Li, 2025), who examined ALBC in conjunction with a traditional Chinese medicinal ointment, revealed a marked decrease in wound healing time, which was consistent with the overall pooled estimate and substantiated the complementary advantages of combined therapeutic approaches.

The underlying mechanism of ALBC’s effectiveness appears to involve establishing elevated local antibiotic concentrations, effectively managing biofilm-associated infections that prove challenging to address with systemic antibiotics alone (Everett and Mathioudakis, 2018). Additionally, when implemented alongside the Masquelet technique, the cement spacer serves to preserve dead space, minimize hematoma development, and potentially facilitate the generation of vascularized membranes (Kavanagh et al., 2018).

Considerable heterogeneity in endpoints like wound healing duration and hospitalization period was anticipated, primarily stemming from clinical differences among patient cohorts (e.g., different Wagner classifications and comorbidities), disparities in ALBC formulation (antibiotic type and dosage), and inconsistencies in concurrent treatments. The initial data discrepancy in the Li et al (Li, 2025). study contributed to this statistical heterogeneity, and its correction enhances the analytical coherence. Conversely, the minimal heterogeneity observed for clinical efficacy rates and amputation incidence bolsters confidence in these specific outcomes.

ALBC’s potential resides in its capacity as an adaptable local delivery system for synergistic combinations. Li et al (Li, 2025). demonstrated that when ALBC was used with Shengji Yuhong ointment, a traditional Chinese medicine preparation known for promoting granulation tissue formation, enhanced therapeutic results were achieved. Moreover, although Zhong et al (Zhong et al., 2024). did not document direct healing metrics, their research offered valuable mechanistic understanding by showing that ALBC combined with negative pressure wound therapy substantially improved granulation tissue development, decreased bacterial colonization, and fostered a pro-healing wound environment marked by M2 macrophage polarization and augmented vascularization. These observations indicate that ALBC functions not merely as a passive antibiotic vehicle but as a versatile localized platform that can be effectively combined with both advanced technologies and supplementary topical agents to orchestrate the multifaceted ulcer healing process.

Our findings align with and extend the conclusions of earlier meta-analyses. Zhao et al. (2024) (Zhao et al., 2024), in a meta-analysis of 21 studies involving 1,409 patients, reported that ALBC significantly improved outcomes, including wound healing (standardized mean difference [SMD] = -1.59) and clinical effectiveness (odds ratio [OR] = 5.26). Similarly, Guo (2025) (Guo, 2025), in a synthesis of 13 randomized controlled trials (843 patients), demonstrated that ALBC significantly shortened wound healing time (mean difference [MD] = -14.46 days) and reduced amputation risk (OR = 0.14). The present meta-analysis, encompassing an updated and larger cohort of 22 RCTs (1,295 patients), not only corroborates these core findings but also provides several novel contributions. First, we performed detailed subgroup analyses by antibiotic regimen, offering further insight into this variable. Second, our study is the first, to our knowledge, to quantitatively synthesize the effects of ALBC on patient-reported pain scores (VAS), demonstrating a significant reduction (SMD = -1.29). Third, we provided a pooled estimate confirming a significant reduction in limb amputation (OR = 0.19). This updated and comprehensive synthesis strengthens the evidence base for ALBC and underscores its multifaceted benefits in DFU management. It is noteworthy, however, that all three meta-analyses, including the present one, are based exclusively on studies conducted in China, highlighting a critical need for international multi-center trials to validate and generalize these findings.

This systematic review revealed a striking geographical pattern, with all eligible studies being conducted only in China. This regional concentration likely reflects unique research priorities or clinical practices within the Chinese healthcare system. While these findings provide robust evidence supporting ALBC application in Chinese patients, they simultaneously underscore a critical void in global research and constrain the external validity of our conclusions. International multicenter randomized controlled trials are urgently required to corroborate and expand upon these observations.

Several methodological constraints warrant discussion. Primarily, certain included studies exhibited suboptimal quality, predominantly due to insufficient documentation of allocation concealment and blinding procedures. Additionally, for several outcome measures, the observed substantial statistical heterogeneity requires careful interpretation of the results. Furthermore, the current evidence base only permits extrapolation to Chinese demographic contexts. Lastly, as previously noted, insufficient data availability prevented meaningful analysis of microbial eradication kinetics. To overcome these limitations, subsequent investigations should implement rigorous blinding protocols and adopt multinational collaborative designs.

## 5 Conclusion

In summary, this meta-analysis confirms that antibiotic-loaded bone cement (ALBC) represents an effective and safe therapeutic option for diabetic foot ulcers (DFU), offering substantial advantages compared to standard wound care. These benefits encompass enhanced wound closure, diminished pain levels, decreased need for surgical interventions, reduced hospitalization duration, and lower incidence of limb amputations. Although limitations exist regarding external validity and blinding procedures, existing evidence justifies ALBC implementation in managing complicated ulcers. Further rigorously designed, multi-center randomized controlled trials across various populations are warranted to consolidate these findings.
