# Supplementary material for: Efficacy and safety of antibiotic-loaded bone cement in the treatment of diabetic foot: a systematic review and meta-analysis
Source: Front Cell Infect Microbiol. 2026 Mar 4;16:1748750. doi: 10.3389/fcimb.2026.1748750 (PMC12996225; doi:10.3389/fcimb.2026.1748750)
Supplement: Supplementary file 1 [file DataSheet1.pdf]

Supplementary Material

Supplementary Figure S1.

Subgroup analysis of hospital stay by Wagner grade.

Supplementary Figure S2.

Subgroup analysis of number of surgeries by antibiotic type.

Supplementary Figure S3.

Sensitivity analysis of VAS score after excluding outlier (Zhang et al.).

Supplementary Figure S4.

Subgroup analysis by antibiotic type for wound healing time.

Supplementary Figure S5.

Subgroup analysis by Wagner grade for wound healing time.

Supplementary Figure S6.

Sensitivity analysis of wound healing time (excluding Wagner grade 5).

Supplementary Figure S7.

Meta-regression of age versus wound healing time.

Supplementary Figure S8.

Meta-regression of diabetes duration versus wound healing time.

Supplementary Figure S9.

Meta-regression of Wagner grade versus wound healing time.

Supplementary Figure S10.

Funnel plot with trim and fill adjustment for wound healing time.

Supplementary Figure S11.

Subgroup analysis of wound healing time by antibiotic type (combination therapy, vancomycin monotherapy, gentamicin monotherapy).

Supplementary Figure S12.

Subgroup analysis of clinical effective rate by antibiotic type (combination therapy, vancomycin monotherapy).

Supplementary Table 1.

Baseline Characteristics and Interventions of Included Studies.

Study ID (Author, Year)	Groups (Sample Size)		Age (Years, Mean ± SD)		Male (n)		Wagner Grade (Range)	Antibiotic Categories
	I	C	I	C	I	C		
Yang2021	17	17	63.0±10.0	-	-	-	2~4	Vancomycin / Gentamicin / Ceftazidime
Han2023	41	41	58.63±5.12	58.68±5.16	23	25	3~4	Vancomycin
Zheng2024	33	33	53.21±5.37	52.24±5.56	19	23	-	Vancomycin / Tobramycin
Wu2021	44	44	55.38±4.12	57.36±2.99	22	23	1~2	Vancomycin
Gao2023	18	18	65.1±5.9	66.2±7.1	10	11	2~4	Vancomycin
Zou2024	56	57	57.33±5.49	58.46±6.98	32	35	-	Vancomycin
Shen2022	20	20	53.14±5.40	53.10±5.45	23	11	2~3	Vancomycin / Tobramycin
Bao2022	32	32	64.04±4.31	63.61±4.82	20	19	4.5	Vancomycin+ Gentamicin
Jiang2024	30	30	63.06±4.25	61.44±3.83	22	20	3~4	-
Cao2023	12	12	64±8	62±8	7	8	2,4	Gentamicin
Zhang2022	44	44	61.35±12.34	61.49±12.52	23	22	1~4	Vancomycin
Liu2021	33	33	-	-	20	19	-	Vancomycin
Li2025	40	40	61.32±4.19	60.13±4.87	18	21	1~2	Vancomycin
Wang2022	25	25	66.10±5.69	65.91±5.84	15	14	-	Vancomycin
Wei2025	35	33	61.35±16.01	64.23±16.31	23	24	2~4	Vancomycin
Huang2023	30	30	-	-	-	-	-	-
Zhang2025	30	30	60.65±9.45	60.25±9.62	17	16	>=3	Vancomycin
Hao2024	26	26	60.77±12.37	58.00±13.66	22	22	2~4	Gentamicin+another one
Zhong2024	12	16	58.83±10.72	67.11±9.25	-	-	3	-

Ehya2021	18	18	48±5.1	45±6.3	13	11	3~4	Vancomycin / Gentamicin / Cefoperazone
Ren2020	20	20	52.6±4.38	51.40±4.99	12	12	3~4	Vancomycin+ Gentamicin
Yang2024	40	20	57.1±8.4	56.5±7.7	21	12	3~4	Vancomycin

Supplementary Table 2.

Reported Outcomes of Included Studies.

Study ID (Author, Year)	Diabetes Duration (Years, Mean ± SD)		Intervening Measure		Reported Outcomes
	I	C	I	C	
Yang2021	-	-	ALBC spacer + VSD	Multiple VSD	①
Han2023	2.89±0.32	2.85±0.29	Modified TTT + ALBC	Modified TTT	②
Zheng2024	9.19±3.02	9.65±2.28	Periosteal Distraction + ALBC	Periosteal Distraction	①,②
Wu2021	4.62±1.13	5.35±1.27	ALBC+VSD	Debridement+VSD	①,②
Gao2023	0.045±0.035	0.052±0.033	ALBC + Dexamethasone Palmitate	ALBC + Normal Saline	①,②,④
Zou2024	-	-	ALBC Coverage	NPWT	①,②
Shen2022	>0.077	>0.077	ALBC + VSD	VSD	①,③,④
Bao2022	0.083±0.016	0.081±0.017	ALBC + Debridement + VSD	Debridement + VSD	①,③
Jiang2024	0.179±0.054	0.208±0.073	Debridement + PMMA Membrane + TCM Ointment	Debridement + VSD + TCM Ointment	①,②

Cao2023	0.151±0.016	0.159±0.016	ALBC (Gentamicin)	Silver Sulfadiazine Cream	①,②,③,⑤
Zhang2022	1.62±0.54	1.58±0.44	ALBC + Debridement	Debridement + NPWT	①,②
Liu2021	0.019~0.110	0.014~0.110	ALBC + Debridement + NPWT	Debridement + NPWT	①,②
Li2025	10.87±2.18	11.13±2.28	Vancomycin ALBC + Shengji Yuhong Ointment	Systemic Antibiotics + Dressing	①,②
Wang2022	0.015±0.006	0.015±0.005	ALBC + Skin Graft	Skin Graft	①,②
Wei2025	14.65±6.42	15.01±5.71	ALBC + Xiaozhong Zhitong Mixture	ALBC	①,②,④
Huang2023	-	-	ALBC + PRP	Conventional Dressing	①,②
Zhang2025	10.55±1.65	10.23±1.58	TTT+ALBC	TTT	①,③,④,⑥
Hao2024	14.73±6.07	13.08±4.78	Dual-antibiotic ALBC + VSD	Debridement + VSD	①,②,③
Zhong2024	0.104（0.069，0.163)	0.149(0.059,0.225)	NPWT+ALBC	NPET	②,B
Ehya2021	-	-	ALBC	VSD	①,③,④
Ren2020	0.109±0.060	0.103±0.058	ALBC+VSD	VSD	①,③
Yang2024	0.08	0.08	ALBC+Conventional Therapy	Conventional Therapy	①,②

Table Note: - , data not available. The original article of Yang2021 reported a total of 21 males across both groups but did not specify the distribution between the intervention and control groups.

Key Abbreviations: ALBC, Antibiotic-Loaded Bone Cement; VSD/NPWT, Vacuum Sealing Drainage / Negative Pressure Wound Therapy; TTT, Tibial Transverse Transport; PMMA, Polymethylmethacrylate ; TCM, Traditional Chinese Medicine; PRP, Platelet-Rich Plasma

Outcome Key: ①, Wound Healing Time; ②, Clinical Effective Rate; ③, Hospital Stay; ④, Number of Surgeries; ⑤, VAS Pain Score; ⑥, Amputation Rate; B, Bacterial Clearance Time (Pre-specified but not analyzed in meta-analysis)
