# Supplementary figures and images for: Efficacy and safety of antibiotic-loaded bone cement in the treatment of diabetic foot: a systematic review and meta-analysis
Source: Front Cell Infect Microbiol. 2026 Mar 4;16:1748750. doi: 10.3389/fcimb.2026.1748750 (PMC12996225; doi:10.3389/fcimb.2026.1748750)

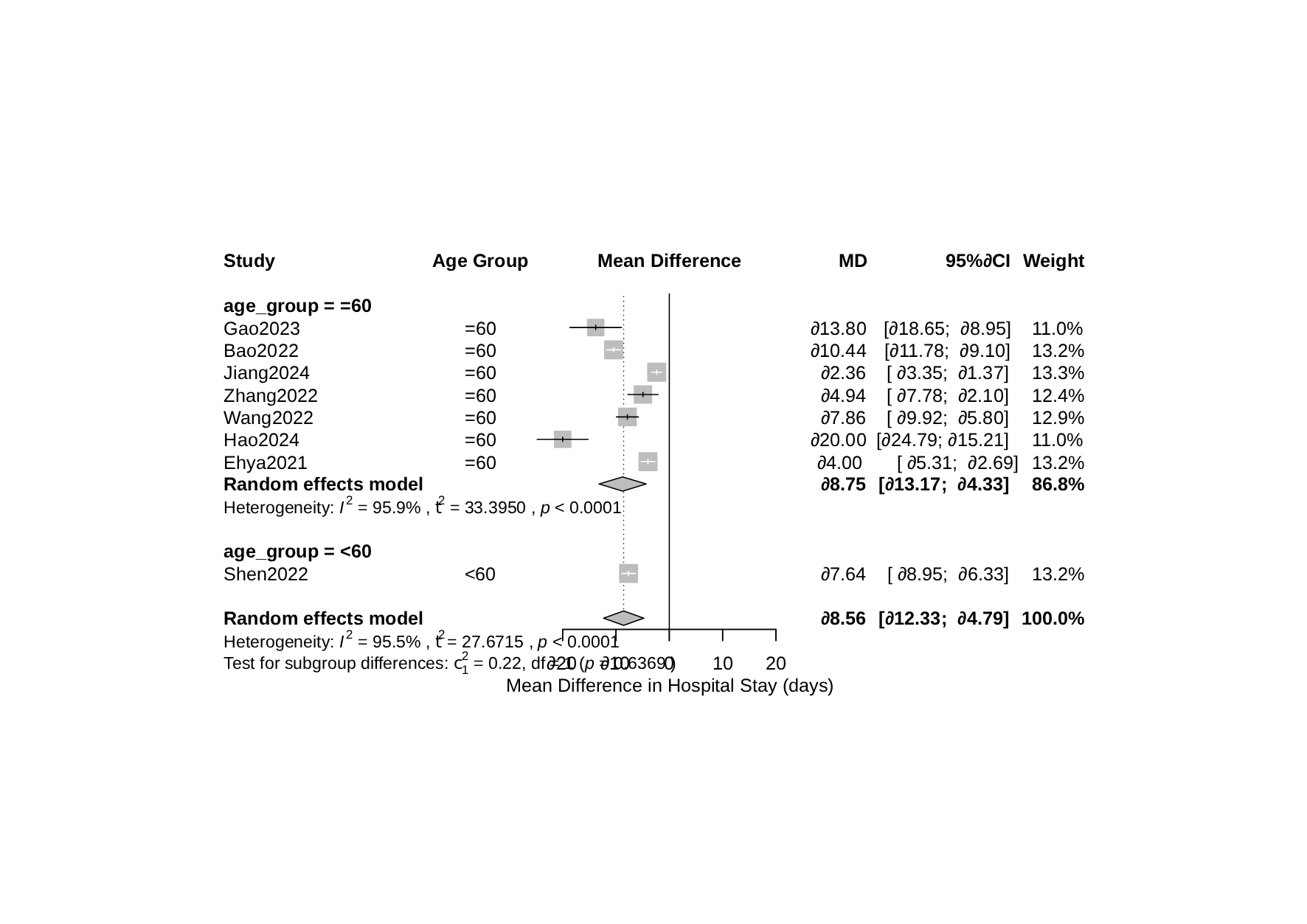

Supplement: Supplementary file 2 [file Image1.tiff]

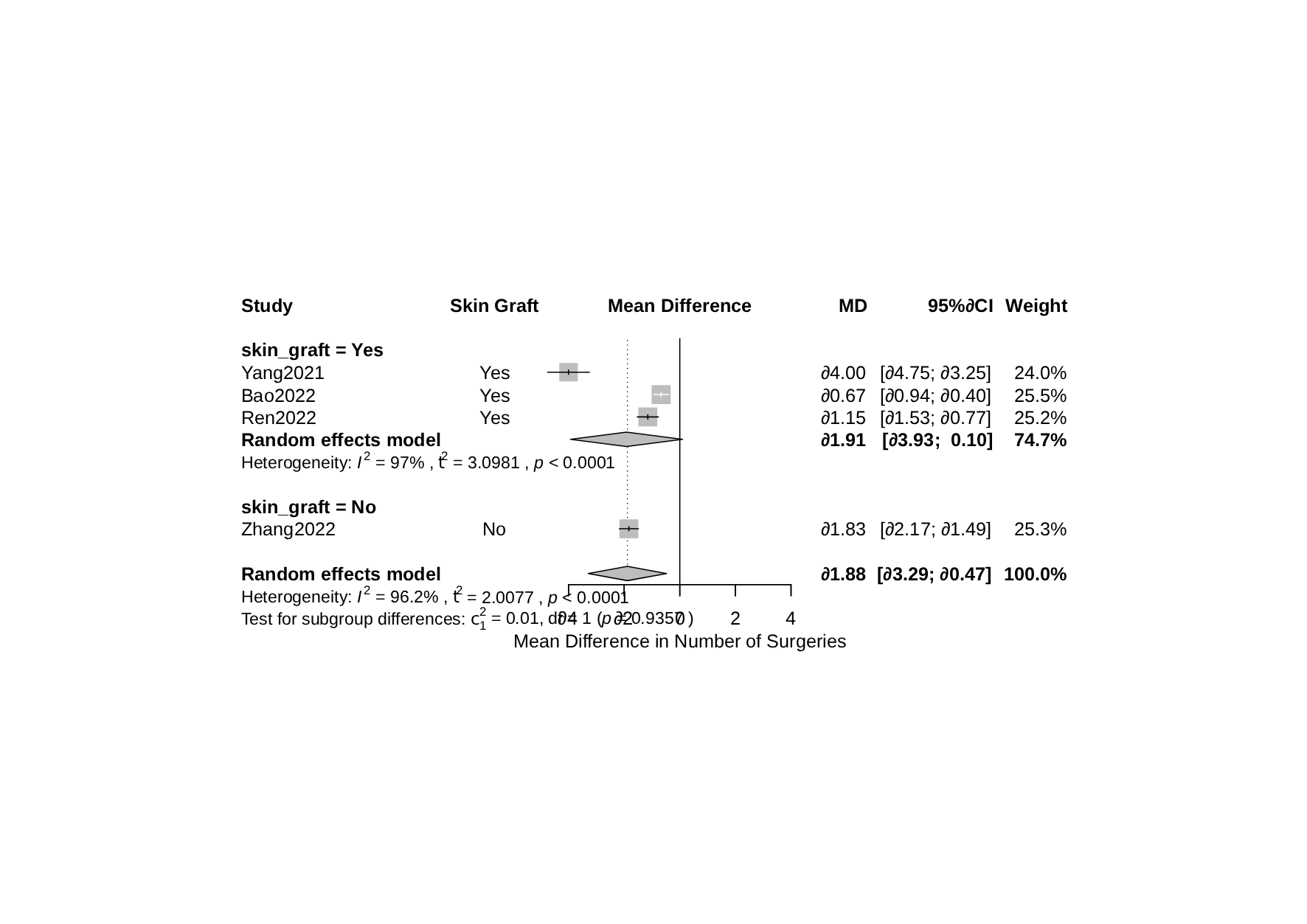

Supplement: Supplementary file 3 [file Image2.tiff]

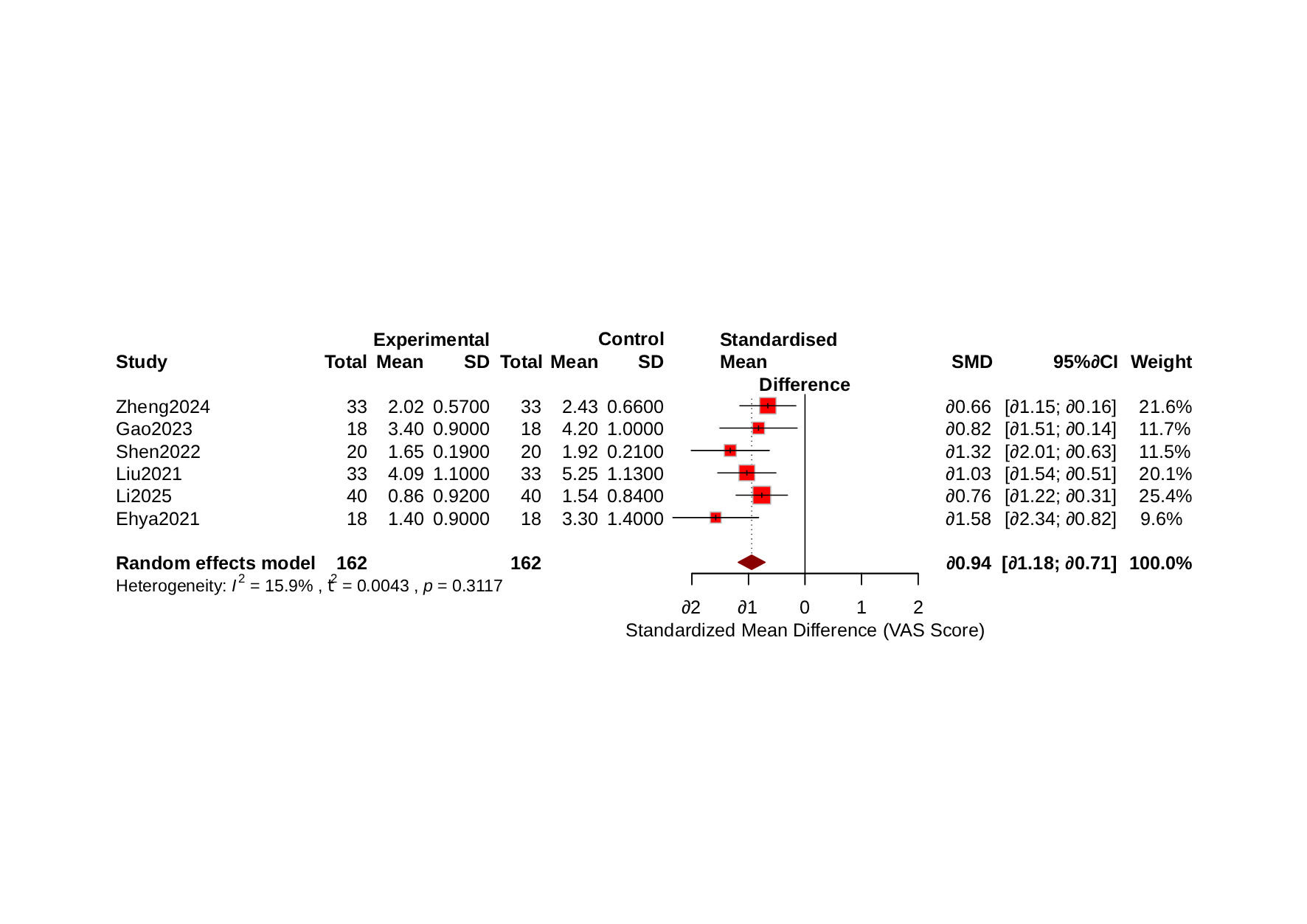

Supplement: Supplementary file 4 [file Image3.tiff]

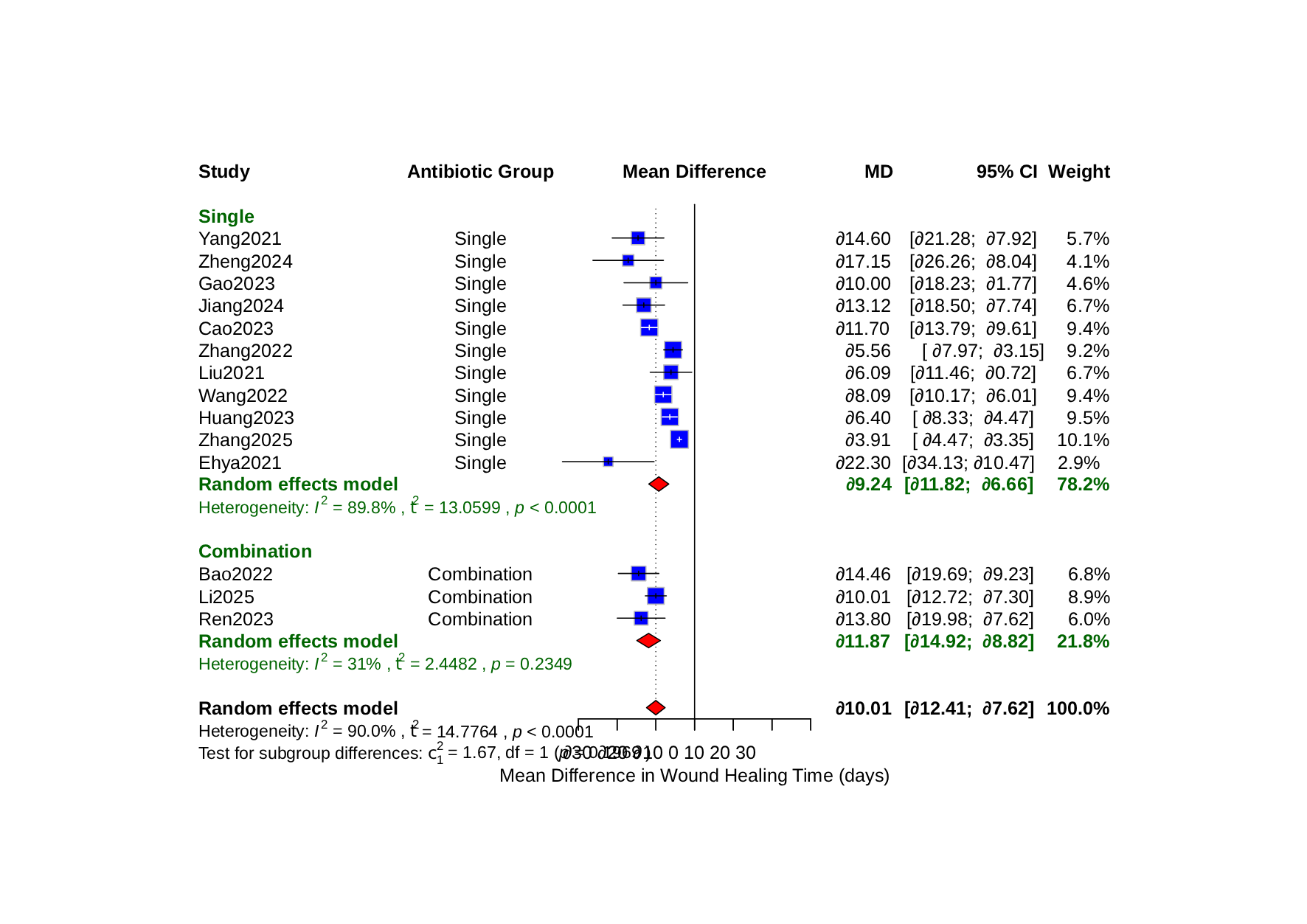

Supplement: Supplementary file 5 [file Image4.tiff]

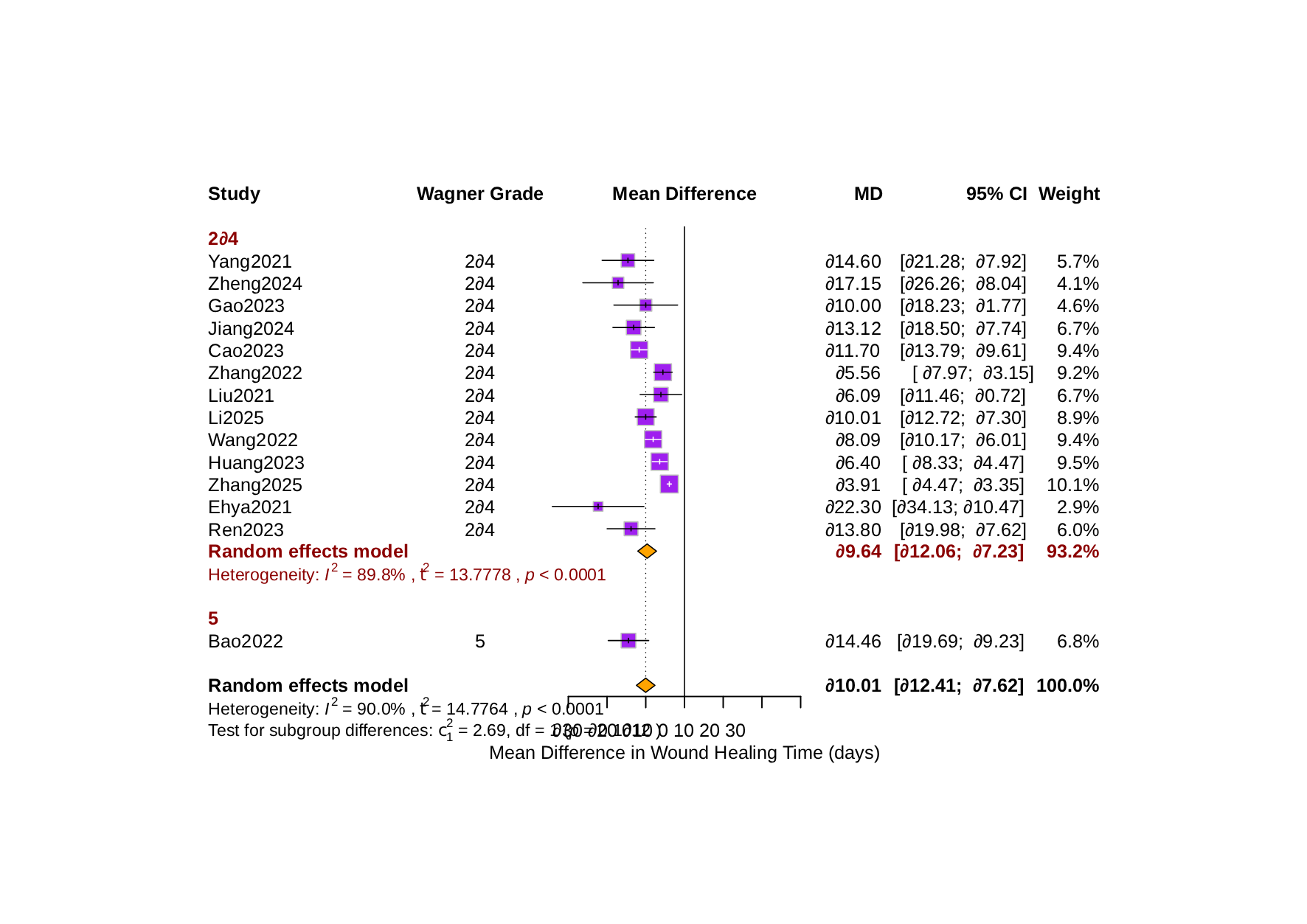

Supplement: Supplementary file 6 [file Image5.tiff]

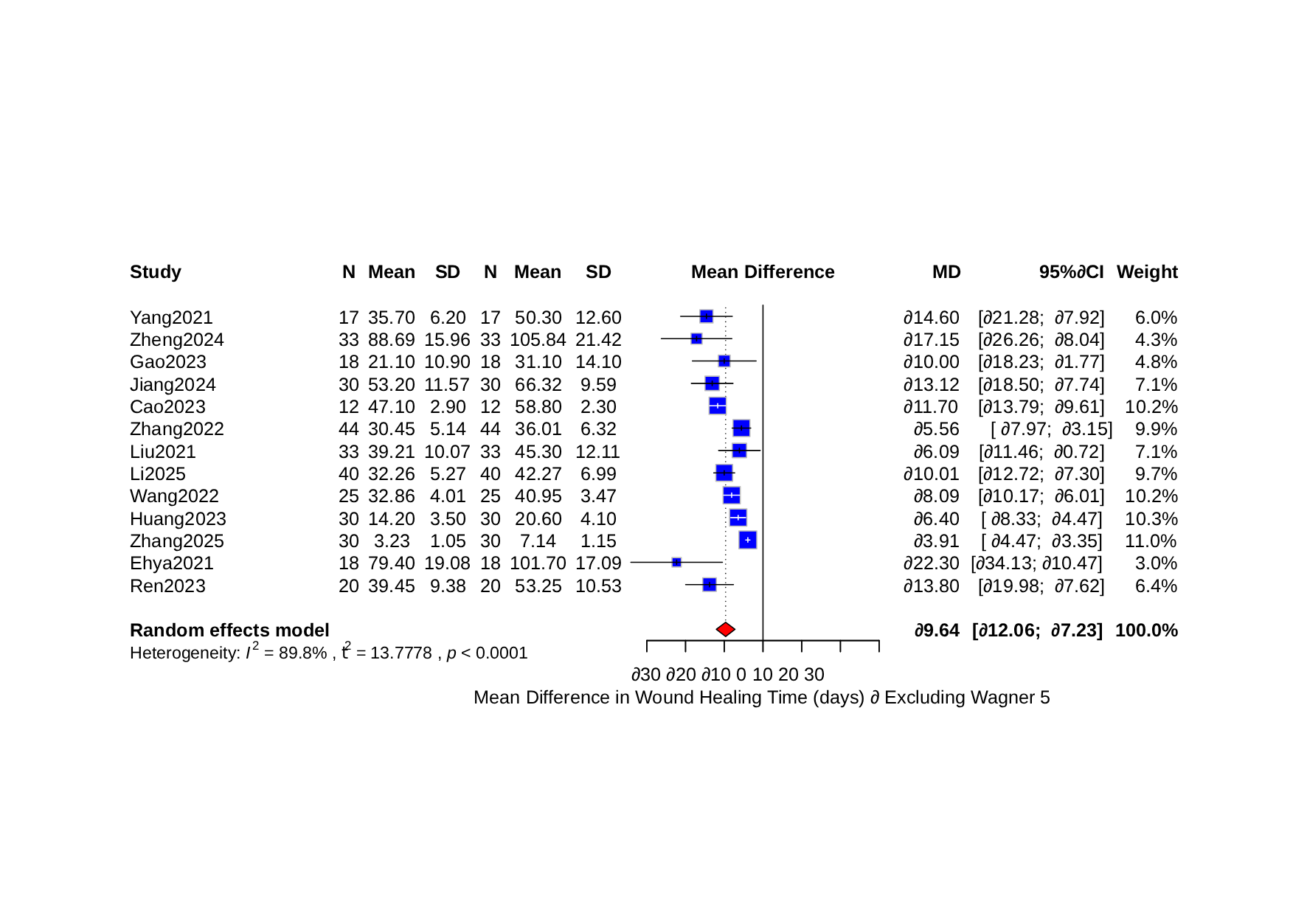

Supplement: Supplementary file 7 [file Image6.tiff]

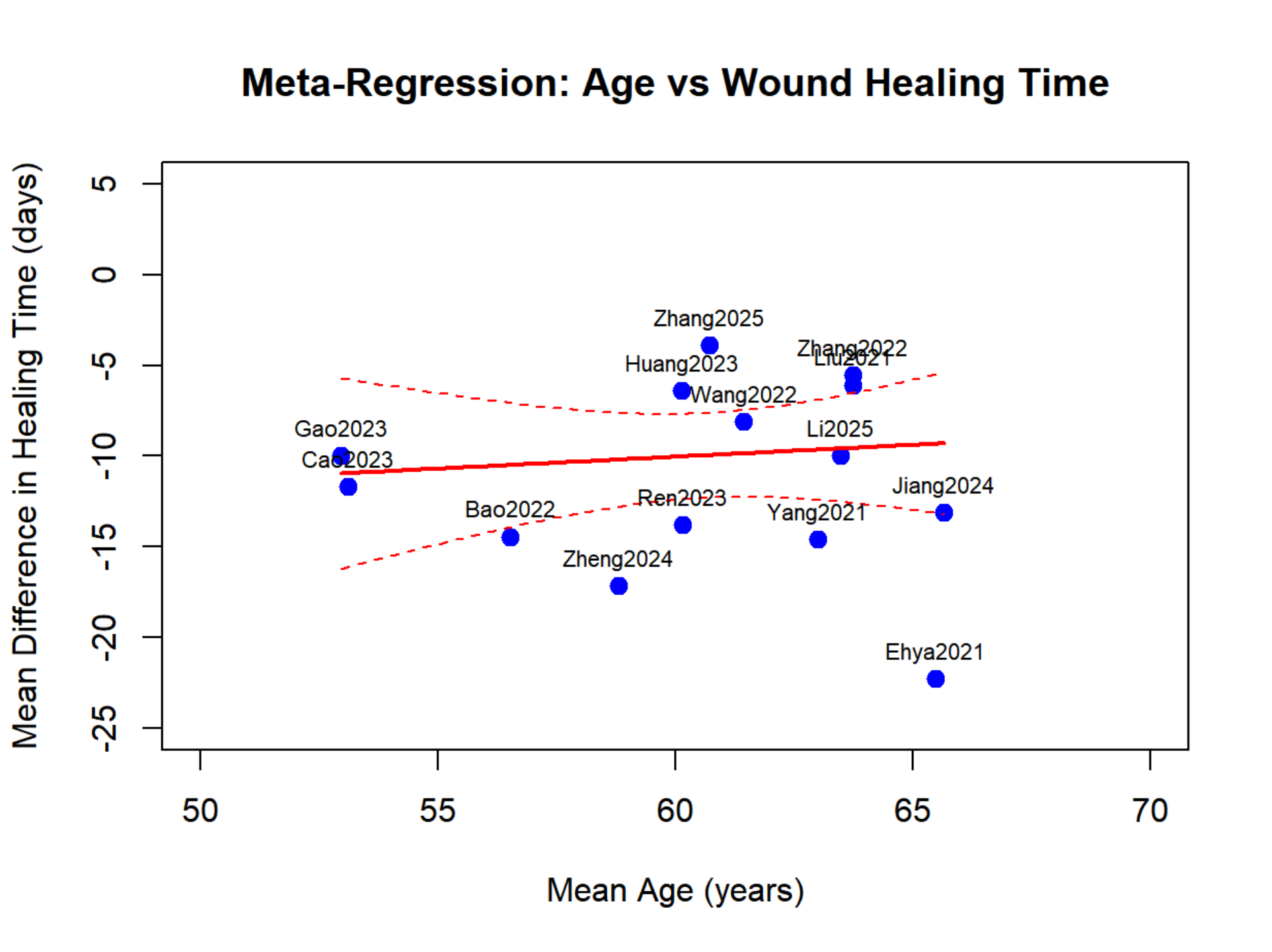

Supplement: Supplementary file 8 [file Image7.tiff]

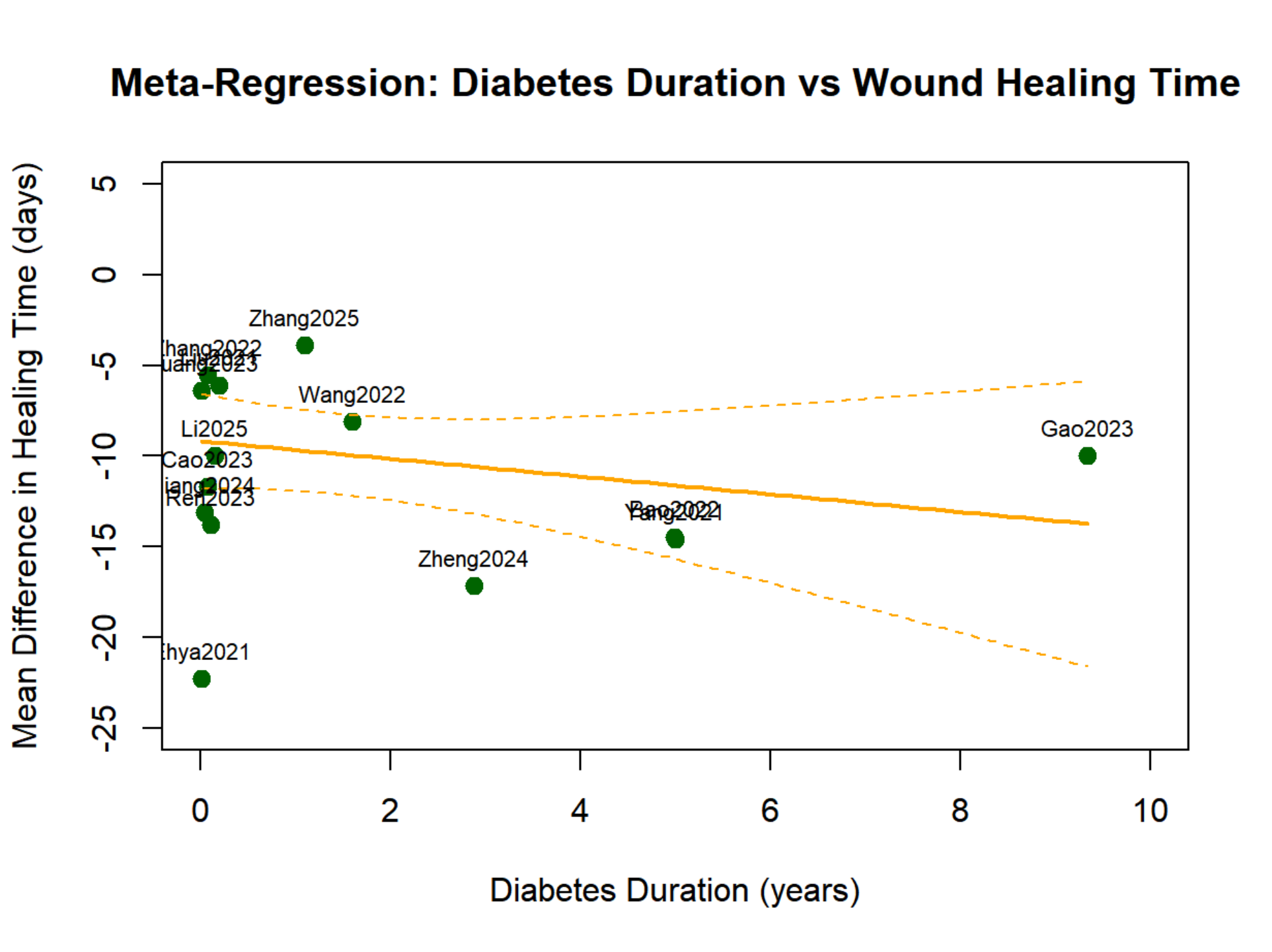

Supplement: Supplementary file 9 [file Image8.tiff]

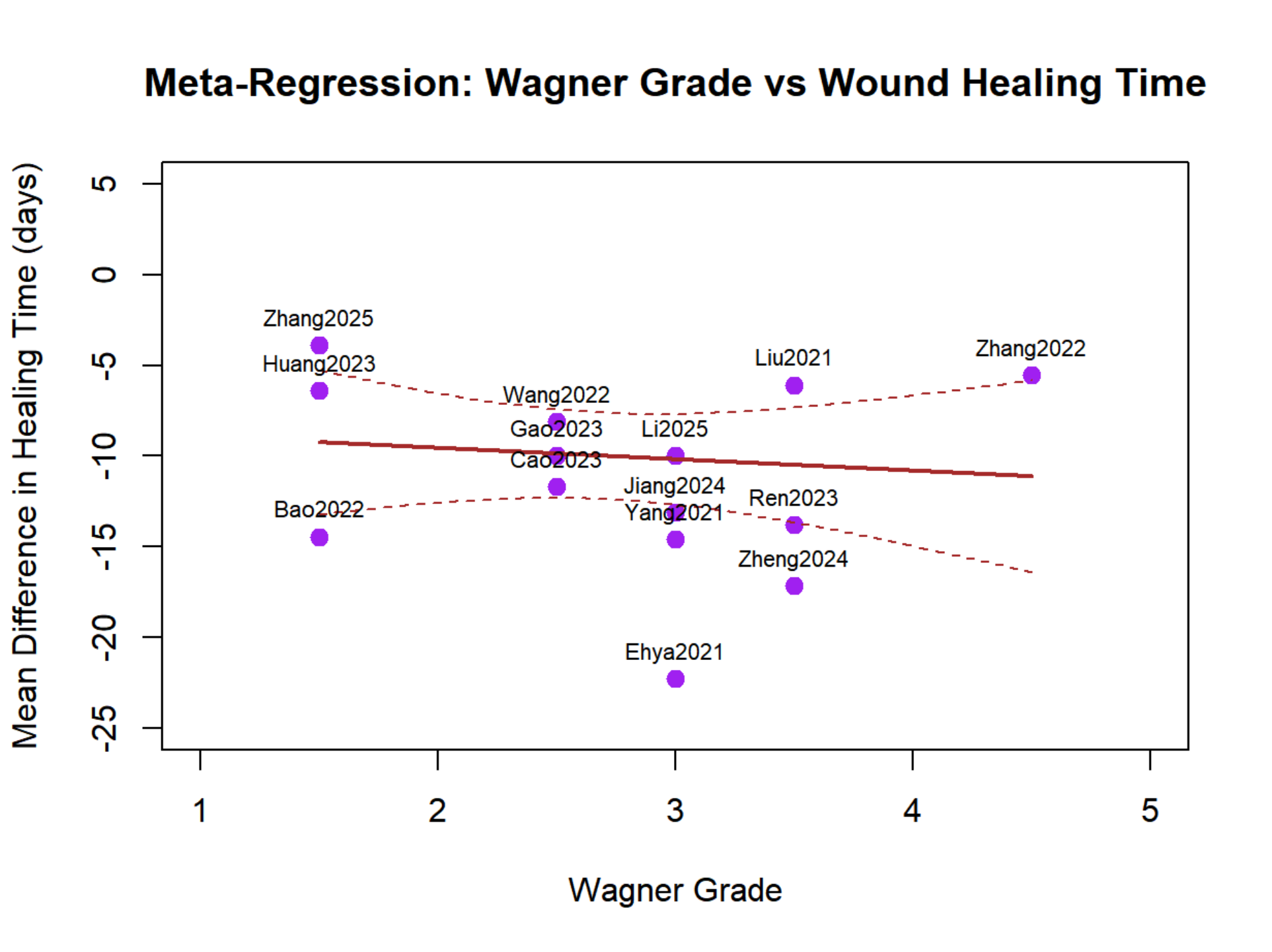

Supplement: Supplementary file 10 [file Image9.tiff]

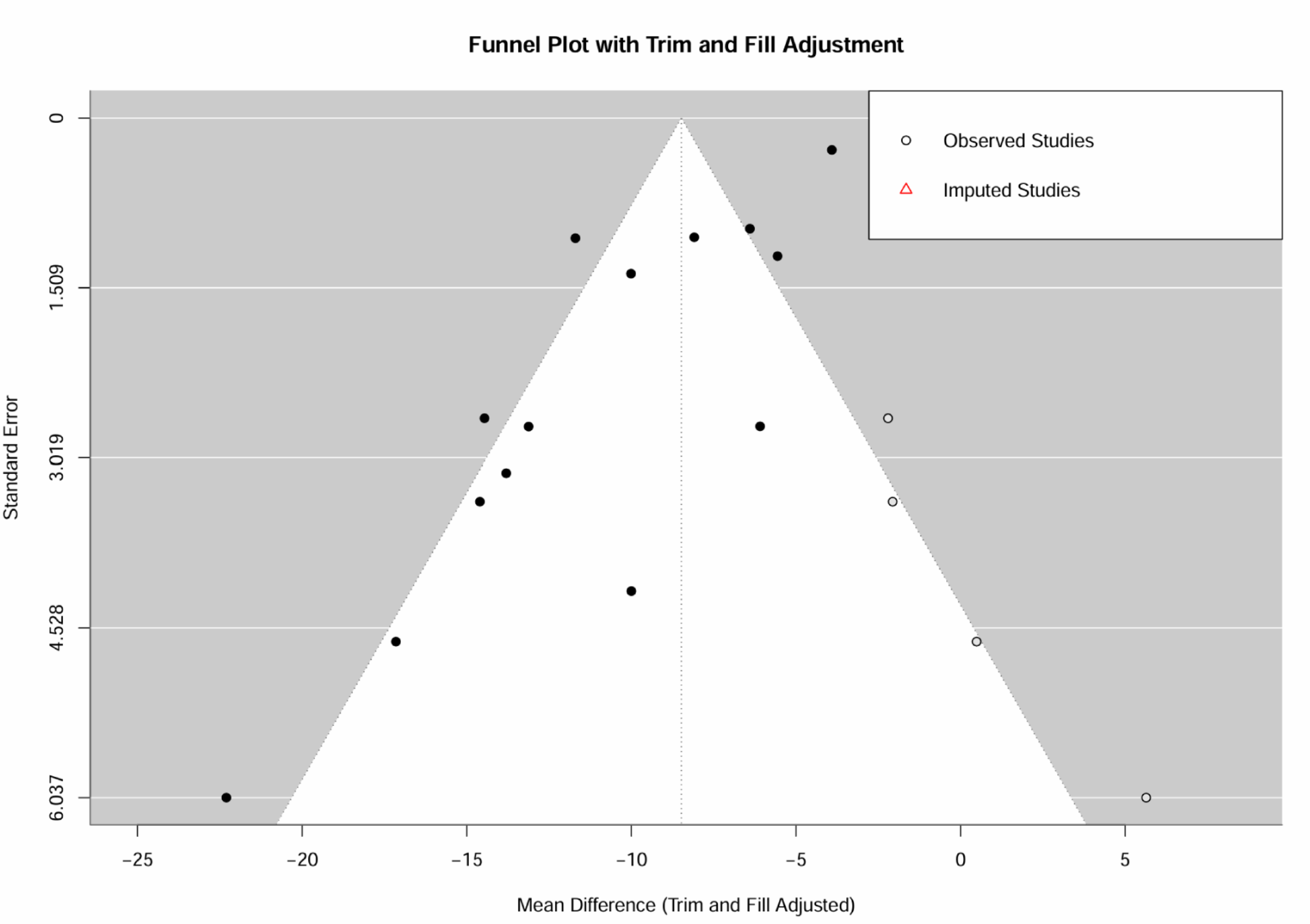

Supplement: Supplementary file 11 [file Image10.tiff]

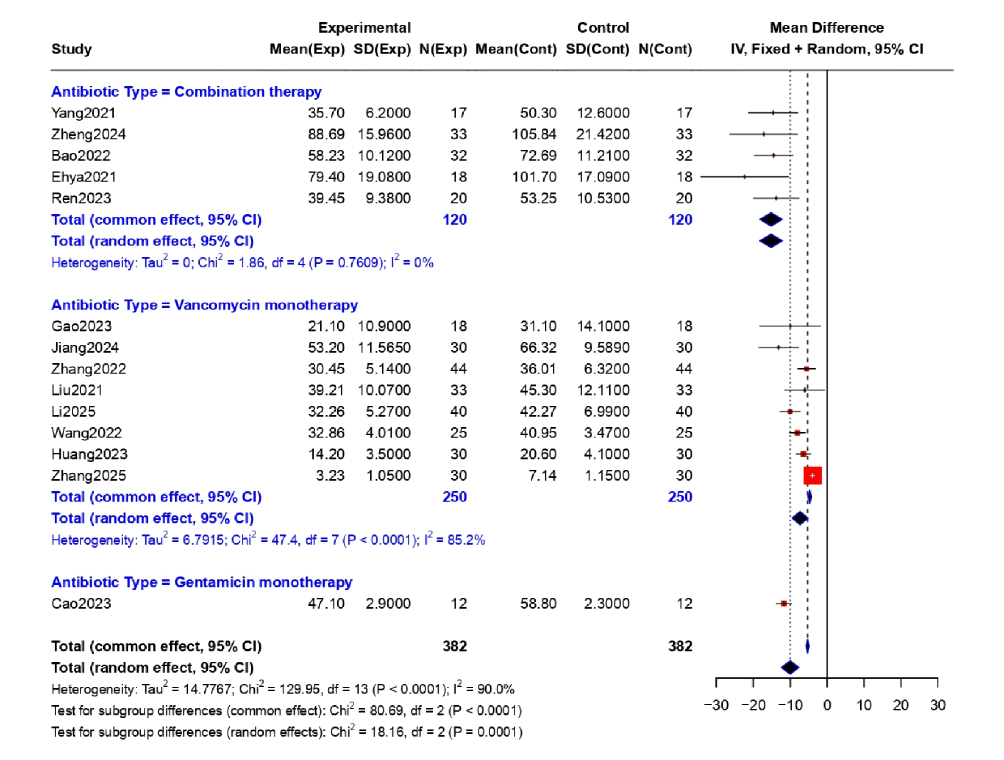

Supplement: Supplementary file 12 [file Image11.tiff]

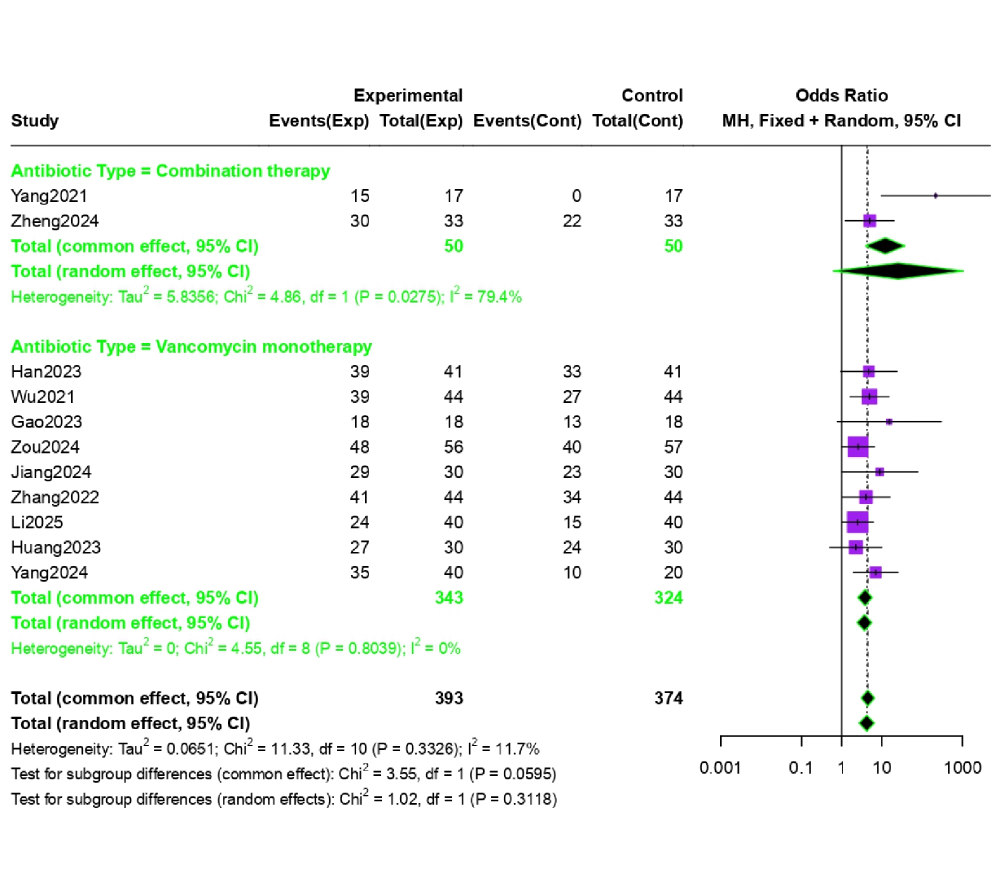

Supplement: Supplementary file 13 [file Image12.tiff]
